# Upper Limb Deep Vein Thrombosis Following Pacemaker Insertion: A Report of Two Cases

**DOI:** 10.7759/cureus.98178

**Published:** 2025-11-30

**Authors:** Chin Kent Lim, Simon Ranjithkumar, Wei Kang Yap

**Affiliations:** 1 General Medicine, Furness General Hospital, Barrow-in-Furness, GBR; 2 Geriatrics, Stockport NHS Foundation Trust, Stockport, GBR; 3 Paediatrics, Barts Health NHS Trust, London, GBR

**Keywords:** dvt investigation, dvt treatment, essential thrombocythaemia, pacemaker complication, permanent pacemaker implantation (ppm), upper limb dvt

## Abstract

Upper limb deep vein thrombosis (DVT) has become a more common condition due to the growing use of cardiac device implantation. We report two cases of left upper limb DVT occurring five days and three months, respectively, after permanent pacemaker implantation. Both patients presented with progressive swelling of the left upper limb associated with dilated superficial veins across the left chest and upper arm. Ultrasound (US) compression venography confirmed occlusive thrombi in the left axillary and subclavian veins. They received direct oral anticoagulant (DOAC) therapy after the diagnosis. This case series highlights the variable timing of symptom onset following permanent pacemaker insertion and emphasises the other risk factors, such as essential thrombocythaemia, to the development of upper limb DVT.

## Introduction

Upper limb deep vein thrombosis (DVT) is relatively uncommon compared with lower limb DVT [1]. However, with the increasing use of permanent pacemakers and implantable cardioverter-defibrillators (ICDs), it has become a more frequently recognised clinical complication [1,2]. The presentation can be classified as acute (occlusion manifesting within days to weeks), subacute (within months), or late (occurring from months to years) following lead implantation [2]. Untreated, it can lead to complications such as a pulmonary embolism (PE) and post-thrombotic syndrome [1]. The pacemaker leads are commonly inserted via the cephalic, axillary, or subclavian veins [2]. Upper limb DVT typically involves the brachial, axillary, or subclavian veins [1]. In our reported cases, both patients had their pacemaker insertion through the left axillary vein, and they developed DVT in their left axillary and subclavian veins.

## Case presentation

Case 1

A 61-year-old man initially presented to the urgent treatment centre with progressive swelling and pain of the left upper limb five days following a dual-chamber (DDD) permanent pacemaker implantation. His past medical history included hypertension, stable angina, and symptomatic second-degree atrioventricular block (Mobitz type 1), for which the pacemaker was implanted. The patient also reported a notable family history, his father’s death in his 50s due to an unprovoked massive PE.

Examination showed the left radial, ulnar, and brachial pulses were present, and the capillary refill time (CRT) was two seconds. The left axillary region was warm to the touch with visibly dilated superficial veins noted over the left brachial area. The entire left upper limb appeared erythematous, markedly swollen compared to the contralateral side and was tender on palpation. A 2 x 2 cm haematoma was observed around the pacemaker site. Otherwise, the pacemaker wound itself was well-healed without evidence of erythema, discharge, or localised warmth. The initial differentials were cellulitis, thrombophlebitis, and a DVT. His National Early Warning Score (NEWS) at presentation was 0 (RR: 17; SpO2: 96% under room air; BP: 153/108; HR: 73; temperature: 36.3; alert). Following this, initial laboratory investigations were carried out (Table 1). 

**Table 1:** Point-of-care testing results

Lab test	Patient’s values	Normal values
Haemoglobin (g/L)	142	125-180
Platelets (x10^9^/L)	175	150-450
White bloods cells (x10^9^/L)	7.3	4.0-10.0
C-reactive protein (mg/L)	12.5	<5

**Figure 1:**
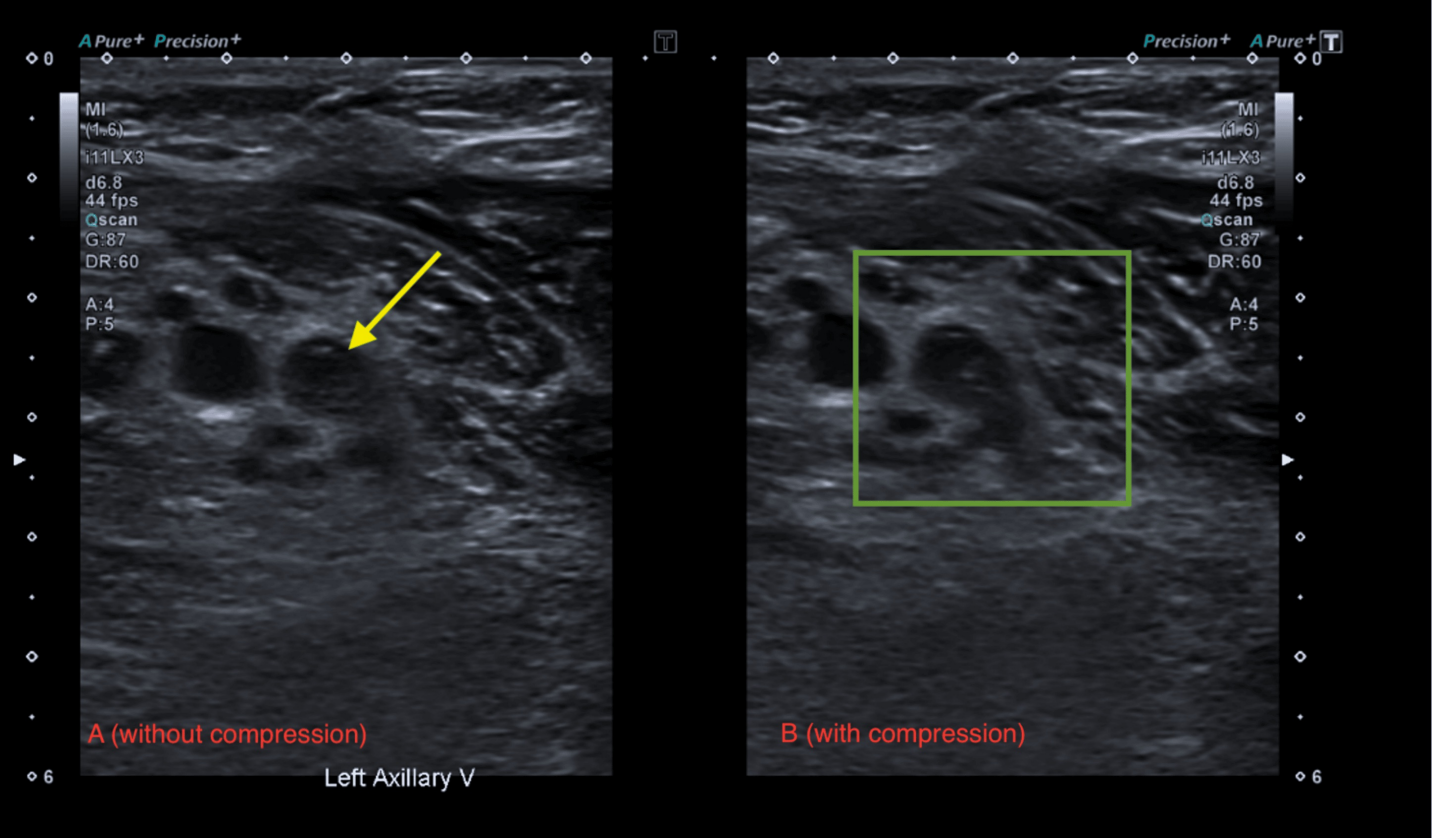
US shows echogenicity (yellow arrow) and lack of compression of the left axillary vein (green box) US: ultrasound (A) US without compression on the left axillary vein. (B) US with compression on the left axillary vein

**Figure 2:**
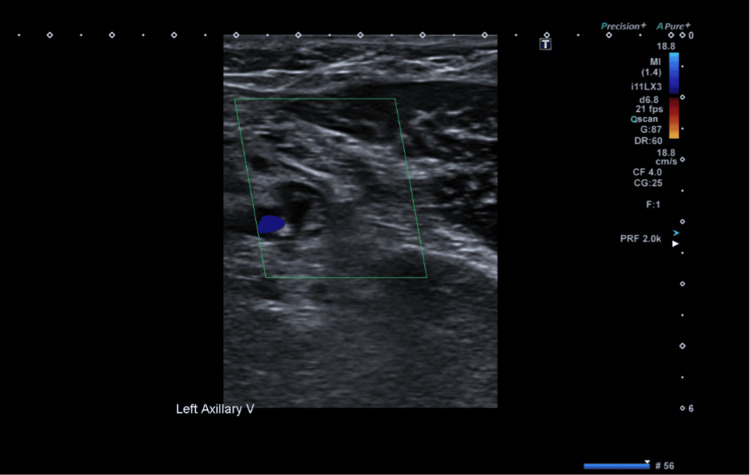
US shows no colour Doppler flow in the axillary vein (green box) US: ultrasound

**Figure 3:**
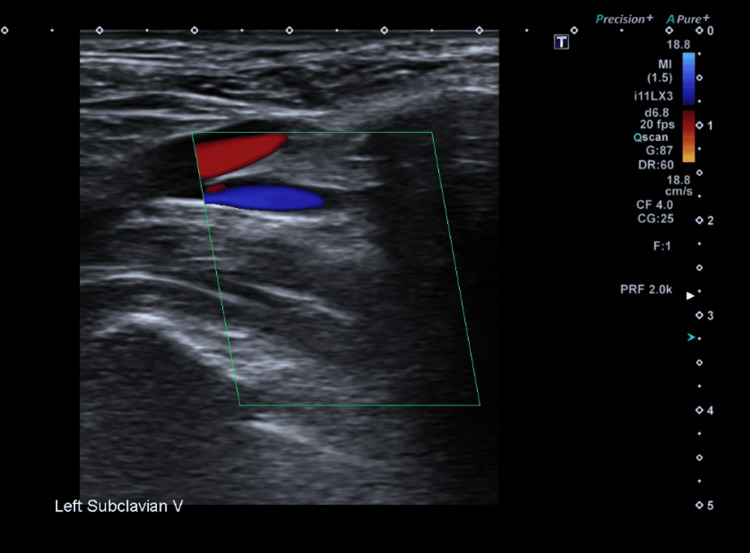
US shows reduced colour Doppler flow in the left subclavian vein (green box) US: ultrasound

A chest X-ray demonstrated satisfactory positioning of the permanent pacemaker leads with no evidence of pneumothorax. Given the patient's clinical picture and the normal inflammatory markers on the preliminary laboratory investigations, a DVT was considered the likely diagnosis. The patient was administered a treatment dose of subcutaneous enoxaparin and was advised to return the following day for an ultrasound compression venography of the left upper limb, as the ultrasound service was unavailable out of hours. The ultrasound confirmed an extensive occlusive thrombus in the axillary vein (Figures 1, 2) and subclavian vein (Figure 3). The brachial vein and internal jugular vein were patent. The patient had an impaired left ventricular function with an ejection fraction of 45-48% noted on a previous echocardiogram, which had been done two months prior to this presentation. No pacemaker checks were carried out during this admission, as the patient was haemodynamically stable throughout. 

The patient was then transferred to the local district general hospital for further cardiology assessment and management. The cardiology team reviewed the patient and recommended a three-month course of anticoagulation for the confirmed upper limb DVT. The patient continued to receive a therapeutic dose of enoxaparin during his hospital stay, and it was switched to apixaban for a further three months upon discharge. His regular aspirin therapy was withheld during the course of direct oral anticoagulant (DOAC) treatment. Follow-up was arranged at the DVT clinic in three months’ time for reassessment and further management as required. Thrombophilia and malignancy screens were performed during admission and were negative. Investigations for a PE were not carried out in the hospital, as the patient did not have any clinical signs such as chest pain, tachycardia, or other haemodynamic instability to suggest this. The patient was then followed up in the DVT clinic in three months, and resolution of the DVT was confirmed. 

Case 2

An 84-year-old man presented with a five-day history of progressive swelling of the left upper limb, also associated with dilated superficial veins over the left chest wall and shoulder. He denied pain, shortness of breath, or chest pain. His past medical history included calreticulin (CALR)-positive essential thrombocythaemia (ET) for which he was taking hydroxycarbamide and aspirin. He developed complete heart block and underwent implantation of a DDD permanent pacemaker three months prior to this presentation.

On examination, the left radial pulse was present with a CRT of three seconds and warm peripheries. There were visibly dilated collateral superficial veins over the left upper chest wall, shoulder, and upper arm. The left upper limb appeared swollen, measuring 33 cm in circumference compared to 28 cm on the right. Pitting oedema was present and extending up to the mid-forearm. An initial differential list included a DVT, cellulitis, haematoma, or thrombophlebitis. His initial NEWS was 0 (RR: 16; SpO2: 96% under room air; BP: 130/74; HR: 67; temperature: 36.5; alert). Preliminary laboratory investigations were carried out (Table 2). 

**Table 2:** Initial laboratory results

Lab test	Patient’s value	Lab value
Haemoglobin (g/L)	126	125-180
Platelet (x10^9^/L)	331	150-400
White blood cell (x10^9^/L)	3.6	4.0-10.0
C-reactive protein (mg/L)	<5	<5
D-dimer (ng/ml)	>1000	0-278

**Figure 4:**
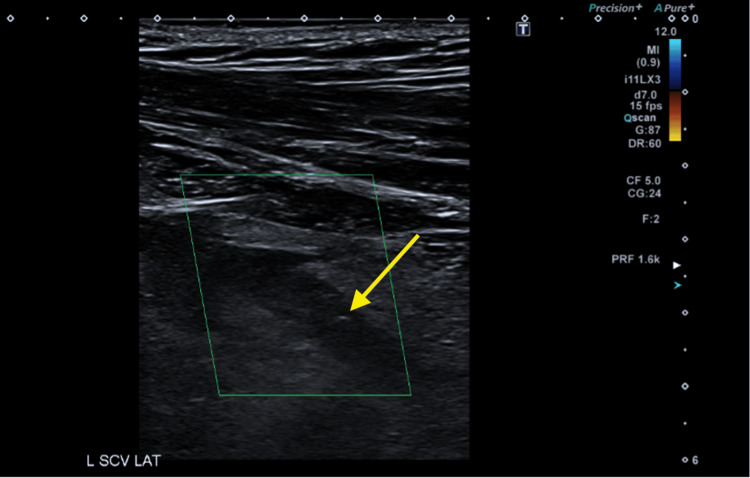
US shows echogenicity (yellow arrow) and lack of compression of the left subclavian vein (green box) US: ultrasound

**Figure 5:**
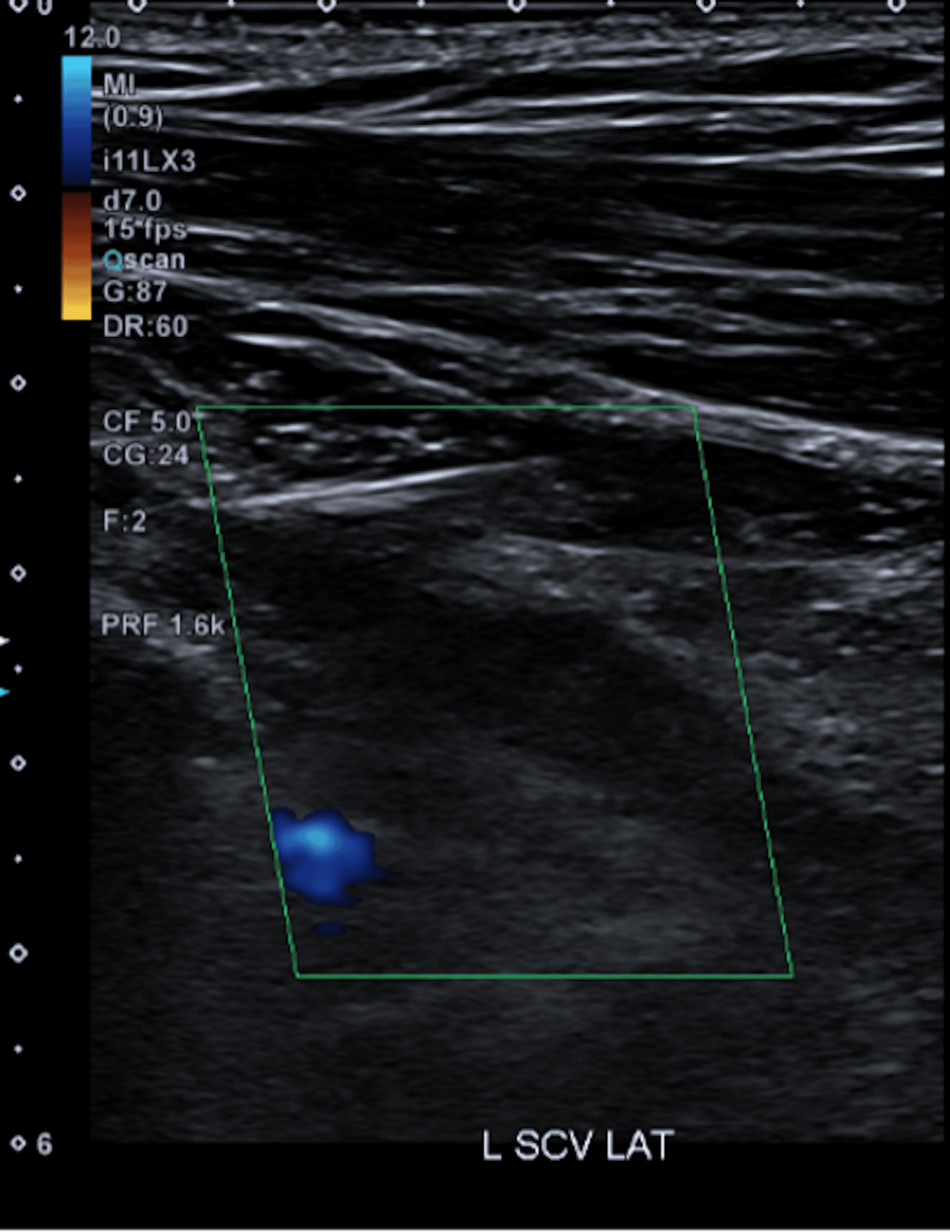
US shows no colour Doppler flow within the left subclavian vein (green box) US: ultrasound

**Figure 6:**
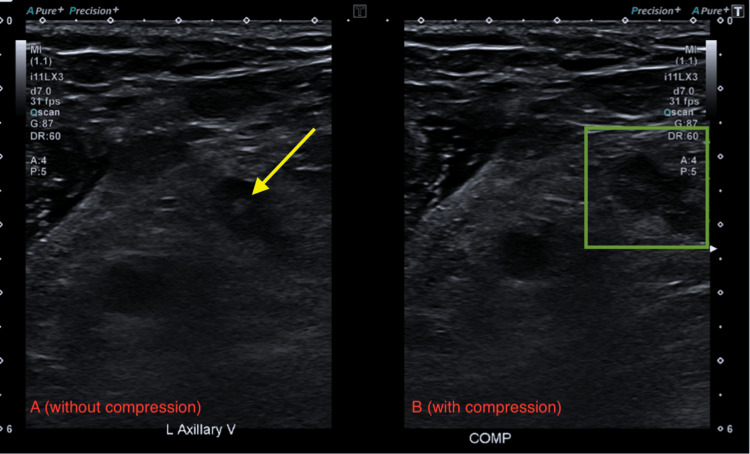
US shows echogenicity (yellow arrow) and lack of compression of the left axillary vein (green box) US: ultrasound (A) US without compression on the left axillary vein. (B) US with compression on the left axillary vein

**Figure 7:**
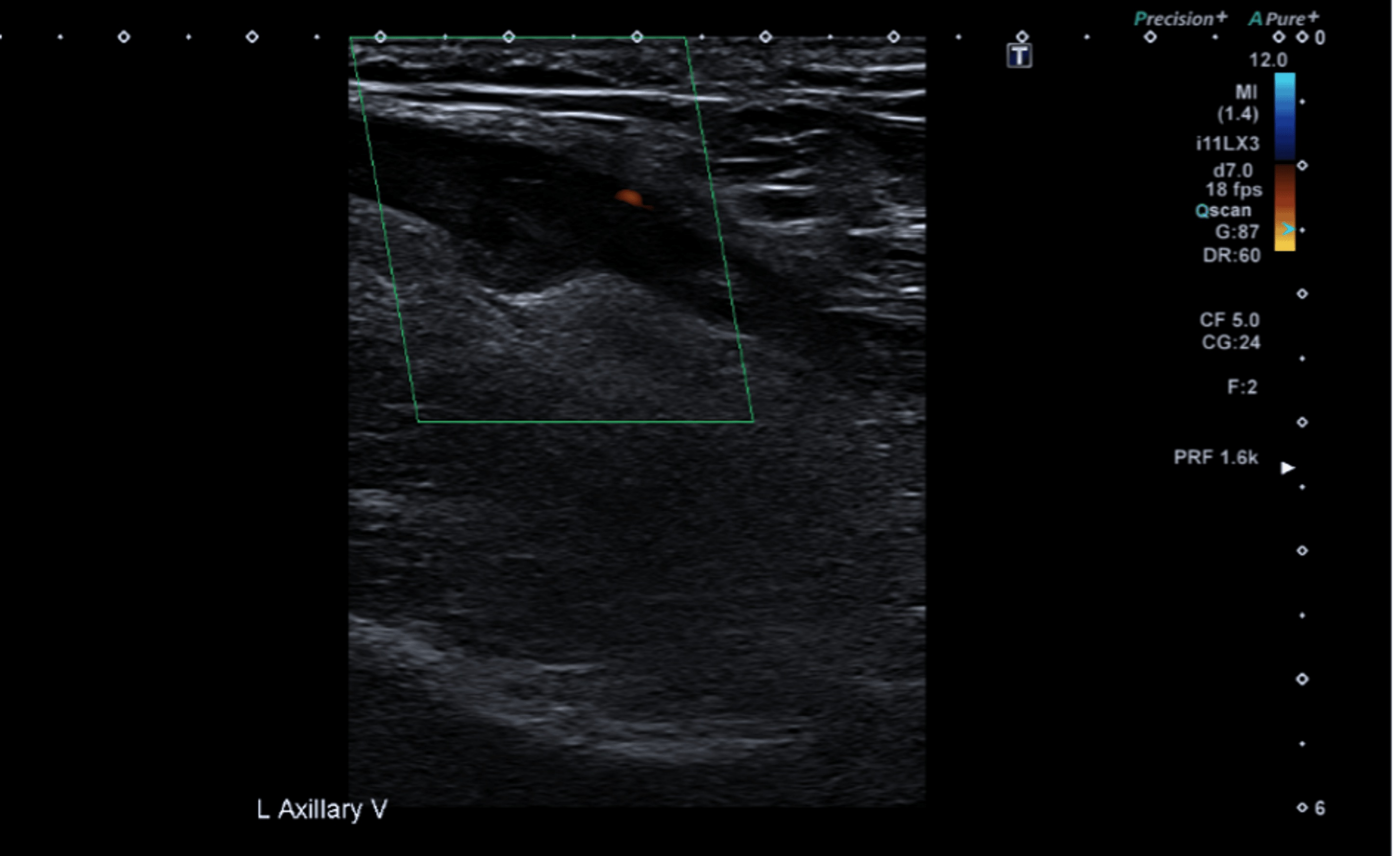
US shows no colour Doppler flow within the left axillary vein (green box) US: ultrasound

Given the patient's clinical picture, the extent of limb swelling, and a positive D-dimer result, a DVT was considered to be the most likely diagnosis in this case as well. US compression venography of the left upper limb confirmed that the left subclavian (Figures 4, 5) and axillary veins (Figures 6, 7) were filled with occlusive thrombus. The left jugular vein was patent with no evidence of thrombus. The patient had an ejection fraction of 65-70% noted on a previous echocardiogram, which had been done three months prior to this presentation. No pacemaker checks were carried out during this admission, as the patient was haemodynamically stable throughout. 

**Figure 8:**
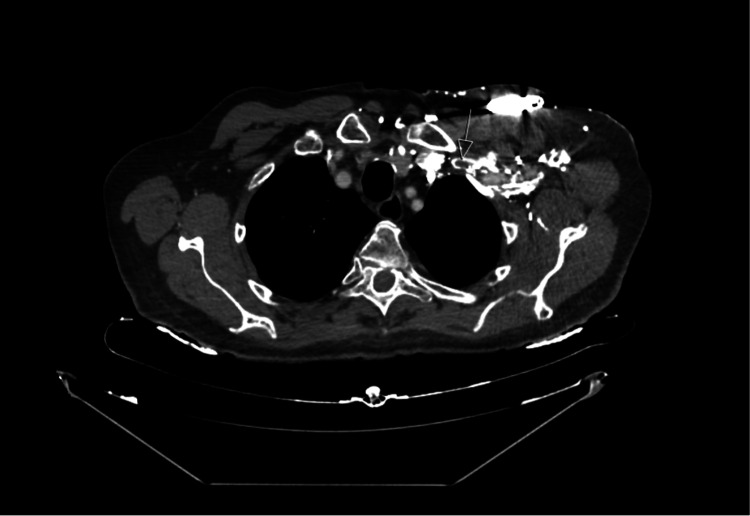
CT TAP confirmed the thrombus within the left subclavian vein (white arrow) CT TAP: CT thorax, abdomen, and pelvis Axial plane

The case was discussed with the haematology team, who recommended a CT thorax, abdomen, and pelvis (CT TAP) and tumour markers to exclude other potential predisposing causes of thrombosis. Tumour marker screening was negative, and CT TAP confirmed the thrombus within the left subclavian vein (Figure 8). No definite malignancy was identified.

The patient was commenced on therapeutic enoxaparin. Then, at the request of the vascular surgical team, the parent medical team discussed the case with interventional radiology, who advised that the clot was thrombosed and collateral venous flow had been formed; therefore, no acute intervention was required. Following the discussion, the vascular surgery team recommended conservative management with anticoagulation. The patient’s therapeutic enoxaparin was subsequently switched to rivaroxaban upon discharge. The patient was then followed up in the haematology clinic in the following month, and symptoms had notably improved. 

## Discussion

The pathophysiology of lead-associated venous occlusion is not fully understood but is believed to involve a combination of thrombosis and fibrosis [2]. In the early stages, insertion of the pacing lead can cause endothelial injury and disrupt normal venous blood flow, thereby activating both the inflammatory and coagulation cascades [1-3]. The endothelial damage and the presence of a foreign body trigger an inflammatory response by releasing neutrophils, macrophages, and foreign body giant cells [2]. In addition, procoagulant factors such as prothrombin fragment, von Willebrand factors, plasminogen activator inhibitor-1, and D-dimer have been shown to increase following lead placement, contributing to a transient hypercoagulable state. Over time, fibrin deposits on the lead surface become incorporated into the intima, associated with vessel wall inflammation. Subsequently, smooth muscle cell infiltration and collagen deposition lead to the formation of a collagen-rich endothelialised capsule around the lead. This progressive process results in venous narrowing and increases the long-term risk of thrombotic events [2].

Other recognised risk factors, such as previous use of a transvenous temporary pacing lead and a left ventricular ejection fraction of less than 40%, have been associated with a higher incidence of venous occlusion within six months following transvenous pacemaker implantation [2,3]. Neither patient in the above cases had an ejection fraction of less than 40%, and they did not require any temporary pacing as they were haemodynamically stable. Additionally, underlying thrombophilia disorders can contribute to the development of venous thromboembolism [4]. In our second case, the patient was an 84-year-old man with CALR-positive ET. ET is a Philadelphia-negative myeloproliferative neoplasm characterised by clonal proliferation of the megakaryocytic lineage within the bone marrow, leading to an elevated platelet count in the peripheral blood and an increased risk of thrombotic events compared with the general population [4]. Following this episode of upper limb DVT, the patient’s advanced age (greater than 60 years) and history of thrombosis placed him at high risk for recurrent thrombotic events. Consequently, the dose of hydroxycarbamide was increased to further reduce the risk of vascular complications.

Ipsilateral arm swelling is typically an early clinical manifestation when the brachiocephalic, subclavian, or axillary veins become occluded [1,2]. However, the swelling may resolve spontaneously as collateral venous flow develops [2]. Over time, the formation of superficial collateral veins across the chest wall and shoulder may become evident. The combination of ipsilateral arm swelling and dilated superficial collateral veins across the chest and shoulder is known as Urschel’s sign [1,2]. Occasionally, the ipsilateral arm swelling can be associated with pain.

These findings should raise suspicion of upper limb DVT, and once venous occlusion is suspected, ultrasound compression venography should be performed as the first-line diagnostic imaging modality [1,2]. It has a reported sensitivity of 97% and specificity of 96% for detecting upper limb DVT [1]. However, assessment of the subclavian vein may be limited due to its anatomical position beneath the clavicle, which prevents adequate compression [2]. In cases where ultrasound findings are inconclusive, CT or MR venography can provide additional diagnostic value and help confirm the diagnosis [1,2].

Upper limb DVT is generally managed with oral anticoagulant therapy such as DOAC for three to six months [2,3,5]. In cases where anticoagulation is insufficient, mechanical interventions such as thrombectomy or thrombus debulking may be considered, but these will need further discussion with the vascular surgeons [2].

## Conclusions

Upper limb DVT secondary to pacemaker or ICD insertion remains an important complication of cardiac device implantation. Venous stasis, endothelial trauma during lead insertion/manipulation, and local inflammatory responses contribute to thrombus formation, particularly in patients with coexisting prothrombotic risk factors. Early identification remains key to improving outcomes, and this is best achieved through a targeted history and examination, followed by appropriate laboratory investigations and imaging, especially in patients presenting with unilateral upper limb swelling, venous distention, or unexplained discomfort in the weeks following implantation, as described in the cases above. DOACs have emerged as a practical and effective therapeutic option for upper limb DVT, offering advantages in terms of ease of administration, absence of routine monitoring requirements, and favourable safety profiles when compared with traditional vitamin K antagonists or heparin-based regimens. In our case, anticoagulation resulted in symptom resolution and preservation of device function, reinforcing their value in clinical practice. In the future, strategies aimed at reducing venous trauma during device implantation and minimising overall lead burden may help mitigate the risk of upper limb DVT. 
